# A Patient With Stage III Locally Advanced Pancreatic Adenocarcinoma Treated With Intra-Arterial Infusion FOLFIRINOX: Impressive Tumoral Response and Death due to *Legionella pneumophila* Infection: A Unique Case Report

**DOI:** 10.3389/fonc.2022.877334

**Published:** 2022-03-31

**Authors:** Girolamo Ranieri, Sara Sablone, Vito Fazio, Dario De Ceglia, Mariangela Porcelli, Pasquale Molinari, Livia Fucci, Carmelo Laface, Cosmo Damiano Gadaleta

**Affiliations:** ^1^ Interventional and Medical Oncology Unit, Istituto di Ricovero a Cura a Carattere Scientifico (IRCCS) Istituto Tumori ‘Giovanni Paolo II’, Bari, Italy; ^2^ Section of Legal Medicine, Department of Interdisciplinary Medicine, Bari Policlinico Hospital, University of Bari, Bari, Italy; ^3^ Histopatology Unit, Istituto di Ricovero a Cura a Carattere Scientifico (IRCCS), Istituto Tumori ‘Giovanni Paolo II’, Bari, Italy; ^4^ Department of Biomedical Sciences and Clinical Oncology, University of Bari Aldo Moro, Bari, Italy

**Keywords:** pancreatic cancer, pancreatic arterial infusion, FOLFIRINOX, arterial port-a-cath, loco-regional treatment

## Abstract

Patients affected by pancreatic ductal adenocarcinoma (PDAC) have very poor prognosis, whereby at a follow-up of 5 years, the mortality rate is very similar to the incidence rate. Globally, around 10% of patients are amenable to radical surgery at the time of diagnosis, which represents the only chance of cure or long-term survival for these patients. Almost 40% of patients with PDAC show locally advanced pancreatic cancer (LAPC). LAPC is not a metastatic disease, although it is not amenable to radical surgery. For these patients, systemic induction chemotherapy with intravenous FOLFIRINOX (5-fluorouracil, folic acid, irinotecan, oxaliplatin) regimen is administered, with the aim of conversion to surgery, although the conversion rate remains low, at approximately 10% to 15%. Pancreatic arterial chemotherapy has been explored to overcome the intrinsic tumor pancreatic resistance to systemic chemotherapy, where an intra-arterial port-a-cath is placed by means of interventional oncology techniques under angiographic guidance in the operating theater. Here, we treated a patient with an intra-arterially modified FOLFIRINOX regimen. Three courses were administered, and the patient experienced no adverse events. At the end of the third course, the patient rapidly developed lung failure due to nosocomial *Legionella pneumophila* infection, despite the impressive pathological tumor response shown in the autopsy report. This is a first and unique report that demonstrates that pancreatic intra-arterial FOLFIRINOX can be safe and efficacious. We believe that this preliminary result will be confirmed in the next patients to be enrolled and that it provides a glimmer of hope for patients with this lethal disease.

## Introduction

Patients affected by pancreatic ductal adenocarcinoma (PDAC) have very poor prognosis, with the mortality rate very similar to the incidence rate at the follow-up of 5 years (1, 2). Globally, around 10% of patients are amenable to radical surgery at the time of diagnosis, which represents the only chance of cure or long-term survival for these patients (3).

Almost 40% of patients with PDAC show locally advanced pancreatic cancer (LAPC) (3), and although this is not a metastatic disease, it is not amenable to radical surgery. This is due to T4 presentation that involves infiltration of the celiac artery, the superior celiac artery, and/or the venous splenic–portal–mesenteric axis, without or with regional lymph node involvement, indicative of stage III according to the TNM classification (8th edition) (4). For these patients, systemic induction chemotherapy with an intravenous (IV) FOLFIRINOX (5-fluorouracil, folic acid, irinotecan, oxaliplatin) regimen is administered, with the aim of conversion to surgery, although with a low conversion rate (10%–15%) (5–7). This low rate is due to several biological and pathological characteristics, among which abundant desmoplastic tissue is a relevant barrier to chemotherapy penetration into the tissue microenvironment (8–17).

To overcome the low chemotherapy efficacy, several studies have explored the possibility to administer intra-arterial (IA) pancreatic infusion chemotherapy through interventional radiology techniques, in particular with gemcitabine, platinum salts, or 5-fluorouracil. These studies have obtained very interesting results in terms of safety, tolerability, and response rates, with low systemic toxicity (18–24). IA infusion chemotherapy has been mainly evaluated in liver colorectal cancer metastases and hepato-biliary cancers, and it is performed in specialized centers with interventional radiology and loco-regional oncological medical expertise in a multidisciplinary team organization (14, 25–27).

The biological background of IA pancreatic infusion therapy is to realize high intra-tumoral concentrations of the chemotherapeutic agents with the longest intra-tumor times, for prolonged drug bioavailability and low systemic toxicity (19). To administer IA chemotherapy, a port-a-cath device is placed into the artery using interventional radiology techniques (21).

## Case Presentation

### First Hospitalization

A 68-year-old woman suffering from recurrent upper abdominal pain in the previous 3 months was hospitalized on December 14, 2020, at the Interventional and Medical Oncology Unit of the National Cancer Institute, Giovanni Paolo II of Bari (Bari, Italy). She had no history of smoking, alcohol consumption, diabetes, known chronic pancreatitis, obesity, or familial cancer. She had a 10-year history of rheumatoid arthritis that was initially treated with prednisone and methotrexate, and then in the previous 2 years, etanercept had been added to obtain better control of the rheumatic disease. For her recent history, she had not shown any vomiting, jaundice, weight loss, or loss of appetite.

Physical examination showed multiple, non-adherent, palpable lateral-cervical, and axillary lymph nodes (maximum diameter, 1 cm). Some swelling of the fingers and wrist joints was also seen. At the time of hospital admission, the patient had undergone a thorax-abdomen computed tomography (CT) scan (October 13, 2020) that had shown a hypodense nodule at the head of the pancreas, with the maximum diameter of 26 mm and an upper abdomen nuclear magnetic resonance (NMR) (October 29, 2020) that had confirmed the pancreatic lesion with a maximum diameter of 25 mm. She also showed carcinoma embryonic antigen and carbohydrate antigen (CA-19.9) in the normal range, with a performance status according to the European Cooperative Oncology Group (ECOG) of 0 to 1.

**Figure 1:**
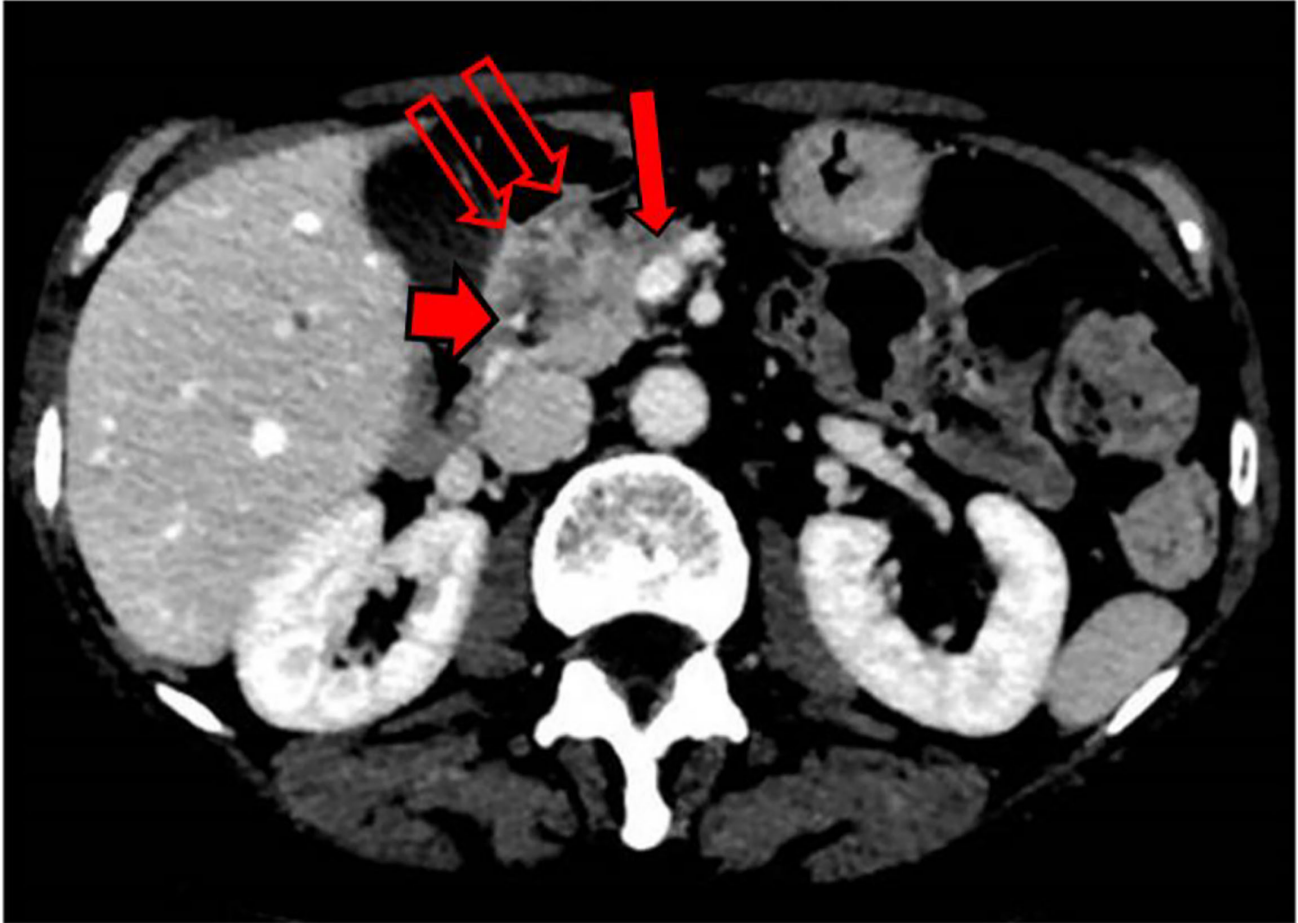
Abdomen CT scan. Single thin arrow, the head pancreatic tumor; short broad arrow, tumor involvement of the superior duodenal-pancreatic artery; twin open arrows, tumor involvement of the portal vein near to the spleno-portal axis.

During this initial hospitalization, the patient underwent ultrasound endoscopy (December 15, 2020), which showed an irregular hypoechogenic head pancreatic nodule with maximum dimensions of 32 mm × 24 mm, and vascular encasement of the gastroduodenal artery and the whole of the superior mesenteric vein. A fine needle biopsy was performed with a gauge 19 needle. A positron emission tomography/CT scan (December 17, 2020) demonstrated a large area of deoxy-glucose uptake in the pancreas (standardized uptake value, 8.4). A total body CT scan (December 18, 2020) showed a lesion at the head of the pancreas with dimensions of 31 mm × 28 mm, with superior mesenteric vein involvement (**Figure 1**).

Further NMR imaging of the upper abdomen (December 22, 2020) confirmed a hypo-vascular tumor with dimensions of 30 mm × 30 mm for the head and the uncinate process of the pancreas. The tumor had infiltrated the superior mesenteric vein, which had resulted in reductions in the circumferences of the portal vein (>180°) and the superior mesenteric artery (<180°). A reduction in the circumference of the inferior vena cava was also seen, with no signs of vascular wall infiltration. Concomitant CA-19.9 assessment indicated an increased value of 163.8 U/ml.

The patient was discharged while awaiting the pathological diagnosis.

### Second Hospitalization

**Figure 2:**
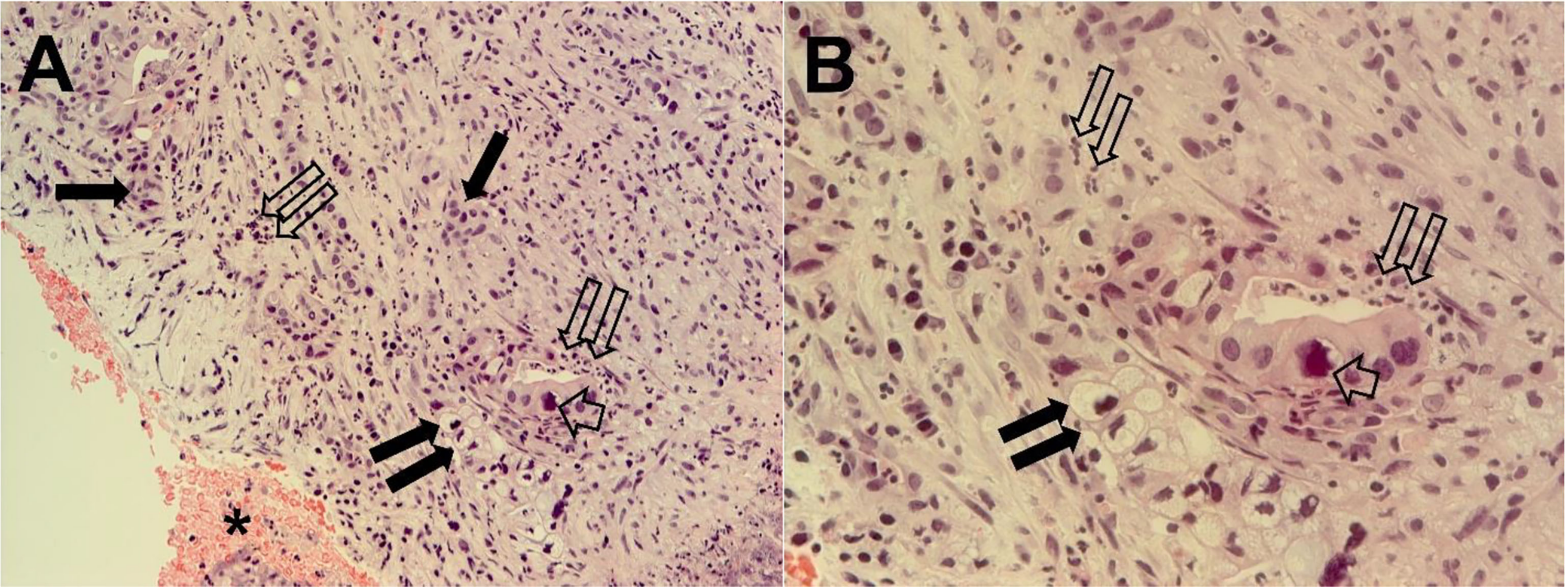
Tumor pancreatic tissue section from ecoendoscopy and needle biopsy cyto included. Tumor cells are organized in differentiated adenomorphic structures or in more poorly differentiated cords, embedded in abundant stroma that are rich in inflammatory cells. Necrotic areas are also evident. Single thin arrows, clusters of tumor cells; short broad arrow, a large nucleus; twin open arrows, inflammatory cells; twin filled arrows, cord of mucous cells with large vacuole and hyperchromic nucleus. The same microscopy field is shown at magnifications of ×200 **(A)** and ×400 **(B)**.

The pathological diagnosis was complete in January 2021, and it revealed a PDAC associated with necrotic and inflammatory phenomena (**Figure 2**). Based on the clinical and pathological diagnoses, the patient was again hospitalized at the Interventional and Oncology Unit of the National Cancer Institute Giovanni Paolo II of Bari.

An updated total body CT scan (January 18, 2021) demonstrated the presence of the known tumor, with 30 mm × 30 mm maximum dimensions and a retro-dilated pancreatic Wirsung duct (diameter, 5 mm). The tumor had further infiltrated the superior mesenteric vein, which had resulted in increased reduction of the circumference (>180°) and also involved the portal vein.

Evaluation of all of the radiology examinations of the PDAC of the patient resulted in its staging as IIIB, according to the TNM classification (8th edition), which was not amenable to radical surgical resection. Due to the low efficacy of systemic FOLFIRINOX as an induction therapy, the patient was offered enrolment in a phase II experimental protocol ongoing at the Institute. The patient accepted the experimental therapy and provided written informed consent to participate in the study.

### Experimental Clinical Study

This experimental clinical protocol was approved in 2020 by the local Ethical Committee of the National Cancer Institute Giovanni Paolo II of Bari (ID N° 948). The study was designed to treat patients with LAPC.

In summary, the study involves infusion of FOLFIRINOX *via* the pancreatic IA route, with the following modified schedule administered on day 1: oxaliplatin 85 mg/m^2^ over 2 h; leucovorin 400 mg/m^2^ over 2 h; irinotecan 130 mg/m^2^ over 90 min; and fluorouracil 2,400 mg/m^2^ as a continuous infusion over 46 h (i.e., starting on day 1). The use of bolus 5-fluorouracil administration was avoided in this schedule (28). Each cycle was for 14 days.

The infusions were carried out using an electromechanical pump to overcome the pressure of the arterial system. To avoid endothelial arterial damage, dexamethasone 8 mg was administered *via* the pancreatic artery before the start of the chemotherapy, and then after the infusion of 5-fluorouracil. Systemic premedication was administered IV with palonosetron 0.25 mg, chlorphenamine 10 mg, and omeprazole 40 mg.

The main inclusion trial criteria were as follows: pathological diagnosis of PDAC; inoperable stage III LAPC; arterial and/or venous vascular encasement by the tumor; ECOG performance status 0 to 1; age, 18 to 80 years; American Society of Anesthesiologists classification from 1 to 3; adequate hematological parameters (hemoglobin ≥9 g/dl; absolute neutrophil count ≥1,500/mm^3^; platelet count ≥100,000/mm^3^); prothrombin time with an international normal ratio ≤1.5 times the upper limit of normal; adequate renal function (serum creatinine, ≤1.5 times the upper limit of normal; creatinine clearance calculated by Cockroft–Gault formula, ≤30 ml/min); adequate hepatic function (aspartate amino transferase, aspartate alanine transferase levels, ≤2.5 times the upper limits of normal; bilirubin, ≤2.5 mg/dl); and written informed consent.

The main exclusion criteria were for metastatic disease, ascites, infected tumor, other previous or concomitant malignant tumors, pregnancy, high risk for no cardiac surgery, presence of metallic stent, HIV, HBV, and HCV infection.

As previously described, the patient was enrolled into this protocol.

### Interventional Technical Procedure

**Figure 3:**
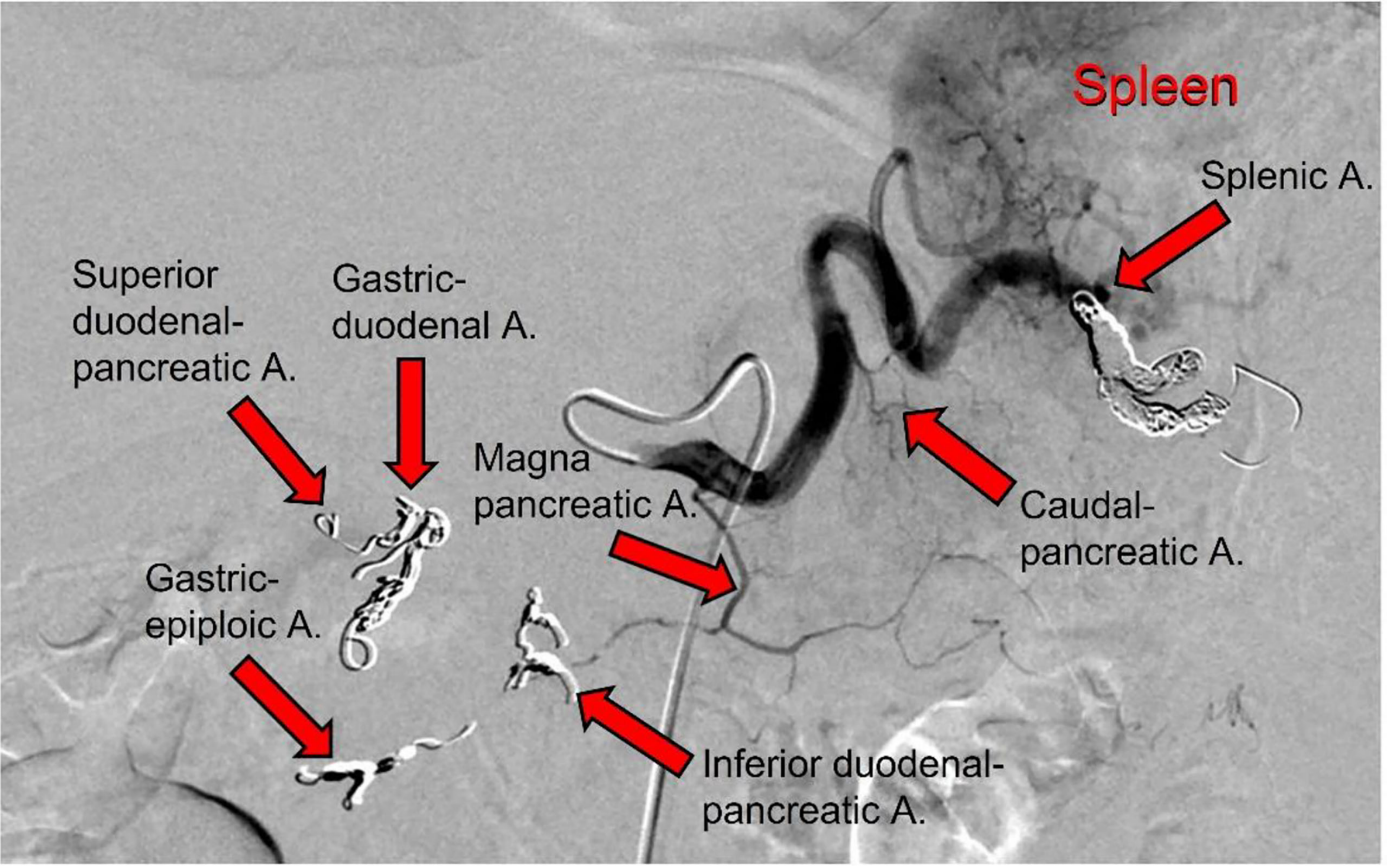
Angiographic scan following injection of contrast medium through the splenic artery after the completion of the technical procedure. The following arteries were embolized, as indicated (arrows): gastric-duodenal a.; superior duodenal-pancreatic a.; gastric-epiploic a.; inferior duodenal-pancreatic a.; and splenica.

During this second hospitalization period for the patient (January 19, 2021), an IA port-a-cath device was implanted. Briefly, in the angio-CT operating theater, under general anesthesia, and using ultrasonic guidance, avascular sheath (6 Fr; Introducer II Standard Kit A; Terumo Radifocus) was inserted into the right femoral artery; this was passed into the aorta artery and then introduced into the celiac axis. Next, microcatheters (2.7/2.4-Fr coaxial microcatheter system; Terumo Progreat) were introduced into the gastroduodenal artery, respectively superior mesenteric artery and splenic artery. All arterial rami were non-pancreatic directed, and the superior and inferior pancreatic duodenal arteries were embolized using magnetic spiral devices (Helix 3D detachable coil system; Axium). This vascular modification was performed to avoid the escape of blood from the pancreas, and in this way the pancreatic arterial system was all sustained by the splenic artery and the ramus from which it originated, including the main great pancreatic artery, which together with the caudal pancreatic artery was anastomosed with the transversal pancreatic artery. Subsequently, the tip of the hydrophilic diagnostic catheter (4 Fr; Glidecath angiographic catheter; Radifocus) was placed at the level of the splenic hilum, and then the hydrophilic guide was inserted into this, to allow the hydrophilic diagnostic catheter to be removed and the definitive placement of the polyurethane catheter (6.5 Fr; PolyFlow polyurethane single-lumen portal; Smiths Medical, Minneapolis, MN, USA) for the IA FOLFIRINOX infusion. At the end of the technical procedure, a selective splenic arterial examination was performed (**Figure 3**).

## Patient Treatment

### First Cycle of IA FOLFIRINOX Infusion

After the interventional procedure, the patient was monitored for several days, with no complications or adverse effects observed. The first cycle of IA FOLFIRINOX was then started on January 26, 2021.

Immediately before starting this IA therapy, the trans-port-a-cath angiography demonstrated the patency of the remodeled pancreatic arterial system. Then, the IA FOLFIRINOX was administered and was seen to be very well tolerated, without side effects and without any hematological grade toxicity.

The patient was discharged from the hospital.

### Second Cycle of IA FOLFIRINOX Infusion, as an Outpatient

The second cycle was administered on February 9, 2021, and again before the therapy, the trans-port-a-cath angiography demonstrated the patency of the pancreatic arterial system. The ECOG performance status of the patient remained at 0 to 1.

Over the following 2weeks, the patient felt good and showed no clinical side effects or hematological toxicity.

### Third and Last Hospitalization

The trans-port-a-cath angiography before the third cycle of the therapy on February 24, 2021, showed thrombosis of the great pancreatic artery. Due to this complication, the patient was hospitalized at the Interventional and Medical Oncology Unit of the National Cancer Institute, Giovanni Paolo II of Bari. She was immediately administered trans-port-a-cat artery thrombolytics, with continuous infusions of urokinase and both IA and IV dexamethasone. The urokinase and corticosteroid infusions were continued, and on February 27, 2021, the trans-port-a-cath angiography indicated partial resolution of the pancreatic artery thrombus. The urokinase and corticosteroid infusions were prolonged, until they were stopped on March 1, 2021, when the trans-port-a-cath angiography indicated the disappearance of the arterial thrombus.

CA-19.9 assessment (March 3, 2021) showed a value of 543.4 U/ml. CT scan evaluation (March 8, 2021) demonstrated a partial response according to the RECIST evaluation criteria (20). This was confirmed by NMR abdomen examination (March 10, 2021), with ECOG performance status well maintained (0-1). On the same day, the trans-port-a-cath angiography confirmed the patency of the arterial pancreatic system, and the third cycle of IA FOLFIRINOX was started.

Unexpectedly, the patient complained of malaise on March 11, 2021, with tachypnea and fast heartbeat. Diagnosis of paroxysmal atrial fibrillation was made, and regression was obtained within 2 h through IV infusion of amiodarone. In addition, on pulmonary auscultation, snores and gasps were evident. Blood gas analysis showed a very low oxygen pressure of 47.7 mmHg. A thorax CT scan demonstrated bilateral pleural effusion, bilateral pulmonary thickening, and ground-glass areas with initial aspects of parenchymal consolidation. Based on radiological findings, the diagnosis of bilateral pneumonia of probable bacterial etiology was made. Oxygen therapy (3 l/min) was added. After infectious disease consultation, broad-spectrum antibiotic therapy was started with piperacillin plus tazobactam 4.5 g IV three times a day, clarithromycin 500 μg IV two times a day, sulfamethoxazole plus trimethoprim 1,920 mg IV three times a day, and ganciclovir 280 mg IV two times a day. Blood cultures and urinary antigenemia for *Pneumococcus* and *Legionella pneumophila* were performed. The patient also underwent bronchoscopy, and the broncho-alveolar lavage fluid obtained was used to investigate cytomegalovirus, SARS-CoV-2 coronavirus, pneumocystis carinii, and common bacteria and fungi.

The result of the urinary *Legionella pneumophila* antigenemia was available on March 12, 2021, at 3:00 p.m., and it was positive. At the same time, the serum search for IgG and IgM toward *Legionella* showed values of 1 (negative) and 40 (positive), respectively. The report of the infectious disease was forwarded to the Italian Health Authorities. Over the day, the patient showed hyperthermia of 38°C.

The continuous IA infusion of 5-fluorouracil was stopped on March 13, 2021. CA-19.9 assessment showed a lower value of 323 U/ml. During the day the patient worsened and showed marked asthenia and dyspnea. Blood gas analysis showed an oxygen pressure of 57.3 mmHg, and oxygen therapy (6 l/min) was applied using a mask (Venturi) over several hours. This provided increased blood oxygen pressure from 80 to 85 mmHg. After resuscitation consultation, continuous positive airway pressure ventilation replaced the oxygen therapy by mask, which obtained an improved blood oxygen pressure of 92 mmHg. In the late evening, the patient worsened again, and blood gas analysis showed a severe hypoxemic blood condition and acute respiratory acidosis.

During the night (March 14, 2021, 12:05 a.m.), the patient was transferred to the post-operatory Intensive Therapy Unit at the National Cancer Institute, Giovanni Paolo II of Bari. The patient was intubated, but at 01:30 a.m., the patient died due to irreversible respiratory failure.

## Autopsy Report

**Figure 4:**
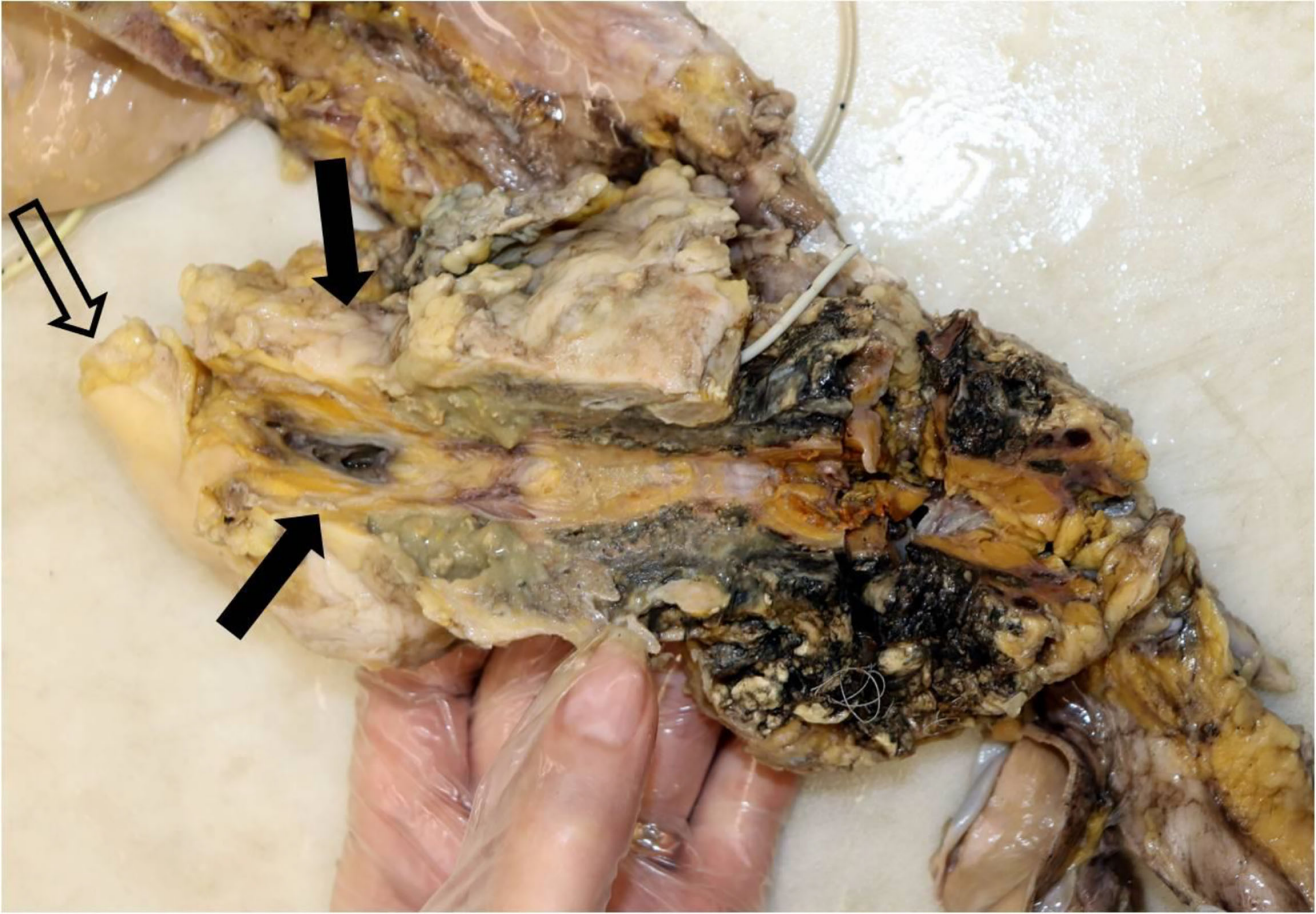
Macroscopic examination of the pancreas, sectioned longitudinally. Single arrows, head of the pancreas, with no visible tumor lesion; twin open arrows, duodenum.

In the subsequent autopsy, the macroscopic pancreas observation indicated head volume reduction with complete tumor mass disappearance (**Figure 4**).

**Figure 5:**
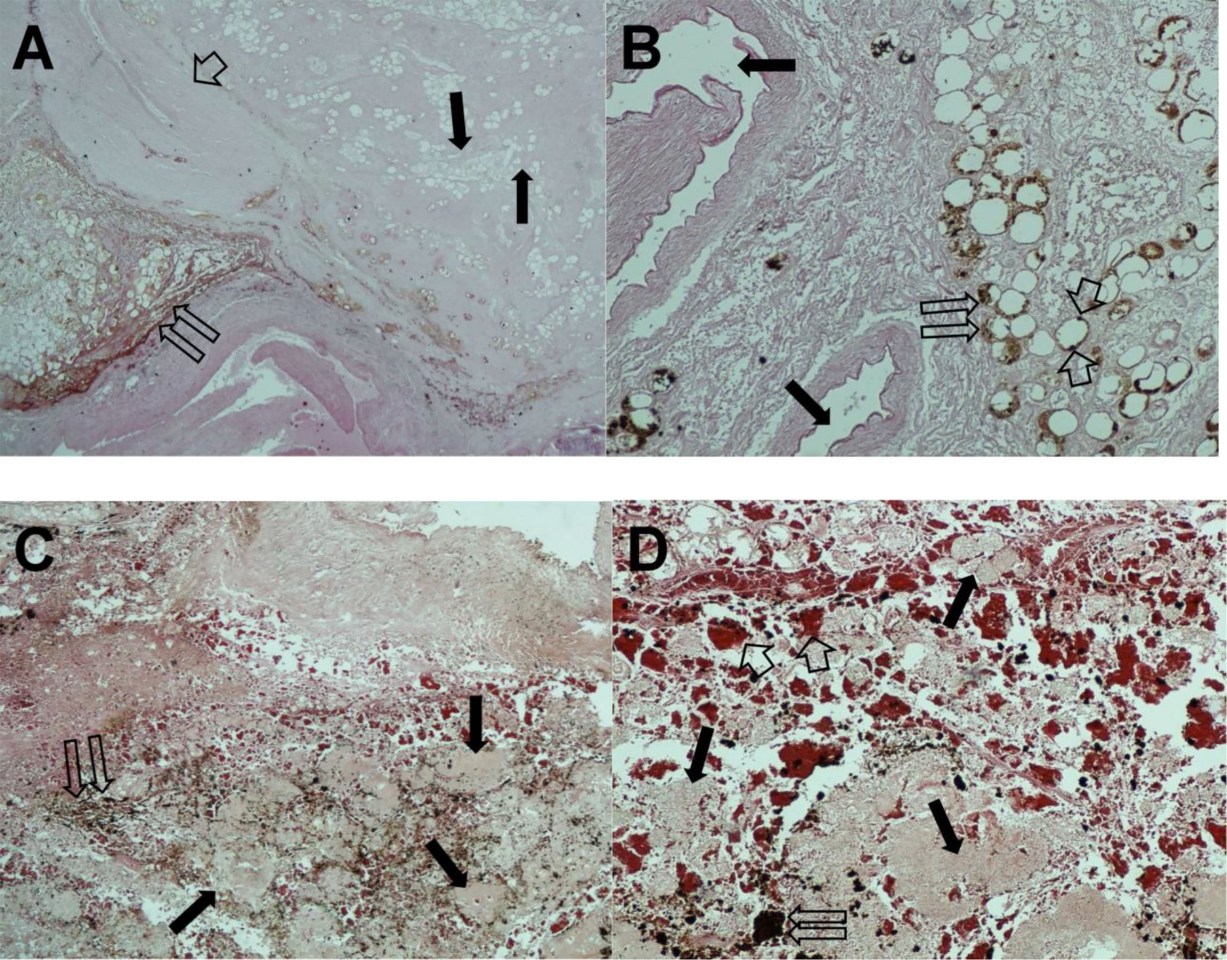
Hematoxylin and eosin staining of pancreatic tissue sections. **(A)** Twin open arrows, necrotic tumor area; single thin arrows, fibrous and adipose replacement; short broad arrow, extensive fibrotic area (magnification, ×100). **(B)** Twin open arrows, residual neoplastic glands with regressive phenomena; single thin arrows, major pancreatic ducts; short broad arrows, single pycnotic nuclei (magnification, ×200). **(C)** The important regressive phenomena. Twin open arrows, hemosiderin deposits; single thin arrows, necrotic tumor area (magnification, ×100). **(D)** Twin open arrows, hemosiderin deposit; single thin arrows, necrotic tumor area; short broad arrows indicate hemosiderin extravasation (magnification, ×200).

**Figure 6:**
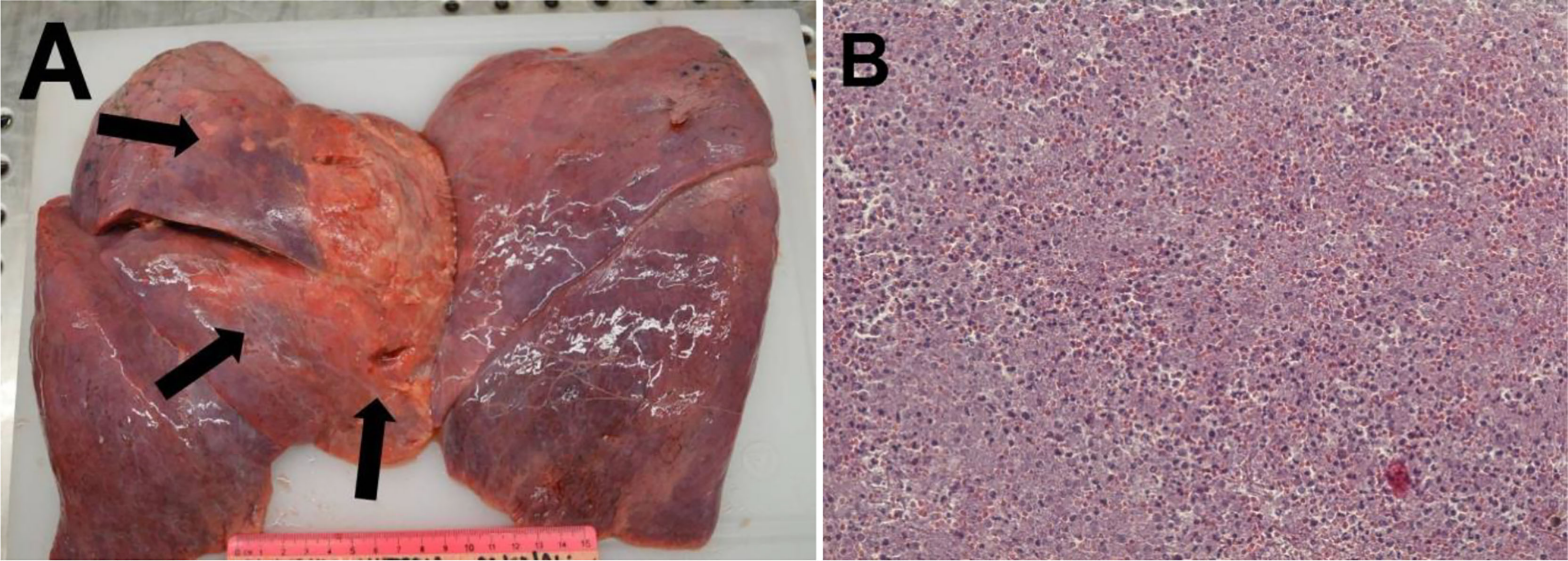
**(A)** Macroscopic examination of the lung. Single thin arrows, surface of the right upper lobe with a yellow-white color. **(B)** Hematoxylin and eosin staining. The completely subverted parenchyma structure with hypoarated or hepatized-like tissue. The alveoli were filled with inflammatory cells, including lymphomonocytes and granulocytes. Red blood cell infiltration is also evident. Features of emphysema can be seen at the periphery of the section, with breaking of the alveolar walls and fusion of the contained spaces.

Histopathological examination of the pancreatic tissue showed complete pathological response for the PDAC, with extensive regressive phenomena of the malignancy associated with an interstitial fibrosclerosis reaction circumscribing the regressed tumoral tissue (**Figure 5**). Here, it is very important to underline that the complete histopathological evaluation of the non-cancerous pancreatic tissue did not show any signs of hemorrhagic–necrotic pancreatitis, which demonstrated that the IA chemotherapy had been safe and that it was not a factor in the induction of pancreatitis. With specific reference to the lung examination, there was a yellow-white color and an increased consistency at palpation particularly on the parietal surface of the right upper lobe (**Figure 6A**). Finally, the pulmonary level showed clear histopathological changes due to severe bacterial *Legionella pneumophila* infection (**Figure 6B**).

## Discussion

Pancreatic ductal adenocarcinoma is the seventh cause of cancer incidence and the fourth cause of cancer deaths in the world (1). This is due to several factors, which include late diagnosis, the intrinsic aggressive biology of pancreatic cancer, and the characteristic desmoplastic microenvironment of the tumor. At a follow-up of 5years, only 10% of patients remain alive (2, 3). This poor prognosis is due to several factors: first, the diagnosis is late in about half of the cases due to a lack of symptoms; and secondly, in the metastatic stage, patient survival ranges from 7 to 11 months with nab-paclitaxel plus gemcitabine or FOLFIRINOX regimens (5, 29).

The only chance to achieve long-term survival for these patients is radical surgery in non-metastatic disease. Globally, surgically treated patients with stage III PDAC plus adjuvant therapy have a median survival of approximately 2 years (30). Here, for patients with ECOG performance status 0 to 1, the FOLFIRINOX schedule is used, while for those with ECOG performance status 0 to 2, the nab-paclitaxel–gemcitabine schedule can be used (31).

Due to the important role of surgery in the therapy of LAPC, the subset of patients with stage III LAPC should be treated upfront with induction chemotherapy, with the aim of downstaging the disease (32, 33). Again, IV FOLFIRINOX or nab-paclitaxel plus gemcitabine are used in the induction setting, although only 10% to 15% conversion rate is obtained, which for induction chemoradiotherapy is a very poor result (6).

To overcome this low response rate to systemic IV chemotherapy, administration of IA pancreatic chemotherapy has been proposed (34–36). The main advantage of IA infusion chemotherapy over IV chemotherapy is that the first-pass effects of the therapy are applied directly to the tumor microenvironment, which produces elevated drug concentrations with greater bioavailability of chemotherapy and few systemic side effects (21).

From a general point of view, the main clinical studies of IA infusion chemotherapy have been performed for liver metastases from colorectal and breast cancers, and primary hepatobiliary tumors (14, 25, 26). These clinical reports have indicated that IA infusion chemotherapy is safe and can provide very encouraging results.

With specific regard to PDAC, the rationale to use IA infusion chemotherapy is even greater considering that the bioavailability of the chemotherapeutic agents near the cancer cells is very low (3). This is due to two main aspects: first, the slender and sparse anatomical pancreatic vascularization, although angiogenic rebound has been demonstrated in PDAC (8, 12); secondly, the abundant dense desmoplastic stromal tissue that characterizes the microenvironment of PDAC, which is a true barrier that is impenetrable to the drugs. Obtaining elevated concentrations of chemotherapeutic agents in the tumor microenvironment might be the prerequisite for the penetration of the drugs into the desmoplastic stromal cancer tissue, to finally arrive near to, and get into, the tumor cells (16).

In the two last decades, it has been demonstrated that IA infusion chemotherapy is feasible and useful in locally and advanced disease using the following drugs: gemcitabine, gemcitabine plus cisplatin, gemcitabine plus oxaliplatin, gemcitabine plus 5-fluorouracil, and epirubicin plus cisplatin (18–23, 35–37). A meta-analysis by Liu et al. (38) indicated the advantages of IA pancreatic infusion chemotherapy over systemic IV chemotherapy (38).

To the best of our knowledge, no report on IA infusion of FOLFIRINOX or modified FOLFIRINOX schedules has been published. Here, for the first time, we treated a patient with LADC with three cycles of modified FOLFIRINOX therapy, after which the CT and NMR evaluations demonstrated radiological tumor response. However, the patient died from acute respiratory insufficiency due to acquired nosocomial *Legionella pneumophila* infection.

Based on the autopsy report and the histopathological report on the non-tumoral pancreatic tissue and the tumoral pancreatic tissue, an impressive pathological complete response had been obtained. On the other hand, no histopathological acute inflammation and acute necrotic hemorrhagic pancreatic reaction was seen for the normal tissue, which demonstrated that this IA FOLFIRINOX infusion therapy was not dangerous for the normal pancreatic tissue.

From a clinical point of view, we would underline the absence of side effects due to the therapy over the three cycles of IA pancreatic infusion, with no hematological toxicities following therapy administration. The particularity of this clinical case is due to the death of the patient due to the indicated nosocomial acquired *Legionella pneumophila* infection, despite the documented pathological tumor response.

This clinical trial is ongoing; however, we feel the need to divulgate this unexpected impressive complete tumor response to the medical scientific community. Finally, we believe that this preliminary result will be confirmed in the next patients to be enrolled, which should thus provide a glimmer of hope for patients with this lethal disease.

## Data Availability Statement

The raw data supporting the conclusions of this article will be made available by the authors, without undue reservation.

## Ethics Statement

The studies involving human participants were reviewed and approved by the IRCCS Istituto Tumori “Giovanni Paolo II” Bari, Italy. The patients/participants provided their written informed consent to participate in this study.

## Author Contributions

Conceptualization, CD and GR. Methodology, GR, SS, and CD. Software, PM, Validation, GR and CD. Formal analysis, GR, CL and SS. Investigation, CD, GR and MP. Resources, CL and VF. Data curation, GR and CL. Writing original draft preparation, GR and CL Writing-review and editing, CL Visualization, PM. Supervision, GR, SS and CD. Project administration, GR, CL and CD. Funding acquisition, PM, VF, DC, and LF. All authors contributed to the article and approved the submitted version.

## Funding

This research is supported by Ministry of Health, Italian Gouvernement, Funds R.C. 2021.

## Conflict of Interest

The authors declare that the research was conducted in the absence of any commercial or financial relationships that could be construed as a potential conflict of interest.

## Publisher’s Note

All claims expressed in this article are solely those of the authors and do not necessarily represent those of their affiliated organizations, or those of the publisher, the editors and the reviewers. Any product that may be evaluated in this article, or claim that may be made by its manufacturer, is not guaranteed or endorsed by the publisher.
